# Survivin inhibition with YM155 ameliorates experimental pulmonary arterial hypertension

**DOI:** 10.3389/fphar.2023.1145994

**Published:** 2023-04-24

**Authors:** Isabel Blanco, Maribel Marquina, Olga Tura-Ceide, Elisabet Ferrer, Ana M. Ramírez, Manuel Lopez-Meseguer, Maria Callejo, Francisco Perez-Vizcaino, Victor Ivo Peinado, Joan Albert Barberà

**Affiliations:** ^1^ Department of Pulmonary Medicine, Hospital Clínic-University of Barcelona, Barcelona, Spain; ^2^ Institut d’Investigacions Biomèdiques August Pi i Sunyer (IDIBAPS), Barcelona, Spain; ^3^ Biomedical Research Networking Center on Respiratory Diseases (CIBERES), Madrid, Spain; ^4^ Biomedical Research Institute-IDIBGI, Girona, Spain; ^5^ Department of Medicine, Addenbrooke’s Hospital, University of Cambridge, Cambridge, United Kingdom; ^6^ Department of Pulmonary Medicine, Hospital Vall d’Hebron, Barcelona, Spain; ^7^ Departament of Pharmacology and Toxicology, School of Medicine, Instituto de Investigación Sanitaria Gregorio Marañón (IISGM), Universidad Complutense de Madrid, Madrid, Spain; ^8^ Department of Experimental Pathology, Institut d’Investigacions Biomèdiques de Barcelona (IIBB-CSIC), Barcelona, Spain

**Keywords:** pulmonary circulation, animal model, hypoxia, SU5416, YM155, pathway

## Abstract

**Background:** Imbalance between cell proliferation and apoptosis underlies the development of pulmonary arterial hypertension (PAH). Current vasodilator treatment of PAH does not target the uncontrolled proliferative process in pulmonary arteries. Proteins involved in the apoptosis pathway may play a role in PAH and their inhibition might represent a potential therapeutic target. Survivin is a member of the apoptosis inhibitor protein family involved in cell proliferation.

**Objectives:** This study aimed to explore the potential role of survivin in the pathogenesis of PAH and the effects of its inhibition.

**Methods:** In SU5416/hypoxia-induced PAH mice we assessed the expression of survivin by immunohistochemistry, western-blot analysis, and RT-PCR; the expression of proliferation-related genes (Bcl2 and Mki67); and the effects of the survivin inhibitor YM155. In explanted lungs from patients with PAH we assessed the expression of survivin, BCL2 and MKI67.

**Results:** SU5416/hypoxia mice showed increased expression of survivin in pulmonary arteries and lung tissue extract, and upregulation of survivin, Bcl2 and Mki67 genes. Treatment with YM155 reduced right ventricle (RV) systolic pressure, RV thickness, pulmonary vascular remodeling, and the expression of survivin, Bcl2, and Mki67 to values similar to those in control animals. Lungs of patients with PAH also showed increased expression of survivin in pulmonary arteries and lung extract, and also that of BCL2 and MKI67 genes, compared with control lungs.

**Conclusion:** We conclude that survivin might be involved in the pathogenesis of PAH and that its inhibition with YM155 might represent a novel therapeutic approach that warrants further evaluation.

## Introduction

Pulmonary arterial hypertension (PAH) is a rare form of pulmonary hypertension that affects small pulmonary arteries. It is characterized by thickened vessel walls that gradually obliterate the arterial lumen and increase pulmonary vascular resistance, which may evolve to right ventricular failure and eventually death (Galiè et al., 2009; Humbert et al., 2010; Galiè et al., 2016).

Vascular remodeling in PAH is mainly characterized by a pro-proliferative and anti-apoptotic phenotype with some similarities to cancer, with sustained increase in proliferation of pulmonary artery smooth muscle cells (PASMC) and endothelial cells (EC), and resistance to apoptosis in vascular cells (Morrell et al., 2001). Since the imbalance between cell proliferation and apoptosis underlie the development of PAH, proteins involved in the apoptosis pathway may play a role in PAH. Survivin (encoded by BIRC5 gene) is a 16.5-kDa member of the inhibitor of apoptosis protein family. It is highly expressed in cancer cells and its expression is associated with tumor progression, proliferation, induction of anticancer drug resistance and poor prognosis (Duffy et al., 2007). Survivin is also expressed in pulmonary arteries of patients with PAH, but not in subjects without PAH (McMurtry et al., 2005), as well as in PASM cells of the monocrotaline-induced PAH model (MCT-PAH) in rats (McMurtry et al., 2005; Lao et al., 2016).

Considering that the survivin signaling pathway plays a fundamental role in cell proliferation and division, it has been proposed that it could contribute to the development of PAH (Blanc-Brude et al., 2002; McMurtry et al., 2005). *In vitro* studies have demonstrated that the inhibition of survivin induces apoptosis and reduces the proliferation of PASM cells (Ai et al., 2006; Ye et al., 2022). McMurtry *et. al*. (McMurtry et al., 2005) also reported that gene therapy with a phosphorylation deficient survivin mutant lowered pulmonary vascular resistance, right ventricular hypertrophy and pulmonary artery medial hypertrophy, and prolonged the survival time in MCT-induced PAH rats. Importantly, serum levels of survivin have been used as biomarkers to assess the operability in patients with congenital heart disease with severe PAH (Li et al., 2019). Overall, these observations suggest that the survivin pathway is disturbed in PAH and could constitute a potential new therapeutic target for the disease.

Survivin expression can be inhibited by administering pro-apoptotic agents such as YM155 (Iwasa et al., 2008; Nakahara et al., 2011). YM155 (sepantronium bromide) is a small, imidazolium-based compound that specifically interacts with the survivin core promoter, inhibiting its gene expression and phosphorylation (Nakahara et al., 2011; Chang et al., 2015). In fact, it has been shown that abrogation of survivin by targeting its transcription with a histone deacetylase inhibitor or directly using a small synthetic inhibitor of survivin was sufficient to promote the apoptosis of arterial smooth muscle cells and to reverse pulmonary hypertension, in both *in vitro* and *in vivo* models of high blood flow-induced PAH and chronic hypoxia (Fan et al., 2015; Lao et al., 2016).

A number of preclinical studies in cancer have reported that YM155 effectively inhibits survivin expression and consequently reduces cell proliferation, increases apoptosis, and sensitizes cells to cytotoxic genes and radiotherapy (Nakahara et al., 2007; Tao et al., 2012). Interestingly, YM155 does not affect normal tissues (Cheng et al., 2016). Phase I and II studies have confirmed the tolerability of YM155 and the achievement of plasma concentrations that induced antitumor activity in patients with advanced cancers (Satoh et al., 2009; Lewis et al., 2011; Tolcher et al., 2012).

The present study aims to further understand the role of survivin in PAH and to assess the effects of treatment with its inhibitor YM155 in the SU5416/hypoxia mouse and in the MCT rat, which are well validated animal models of PAH. We assessed survivin expression in both the SU5416/hypoxia mouse and the MCT rat models (Blanco et al., 2017). In the SU5416/hypoxia mouse model (Bueno-Beti et al., 2018) we additionally assessed the effects of its inhibition with YM155 on pulmonary hemodynamics, pulmonary vascular remodeling, RV hypertrophy, and cell apoptosis and proliferation. The expression of survivin and its relationship with apoptosis/proliferation pathways was further assessed in explanted lungs from patients with PAH.

## Materials and methods (more details are provided in the online supplemental material)

### *In vivo* models of pulmonary hypertension

**FIGURE 1:**
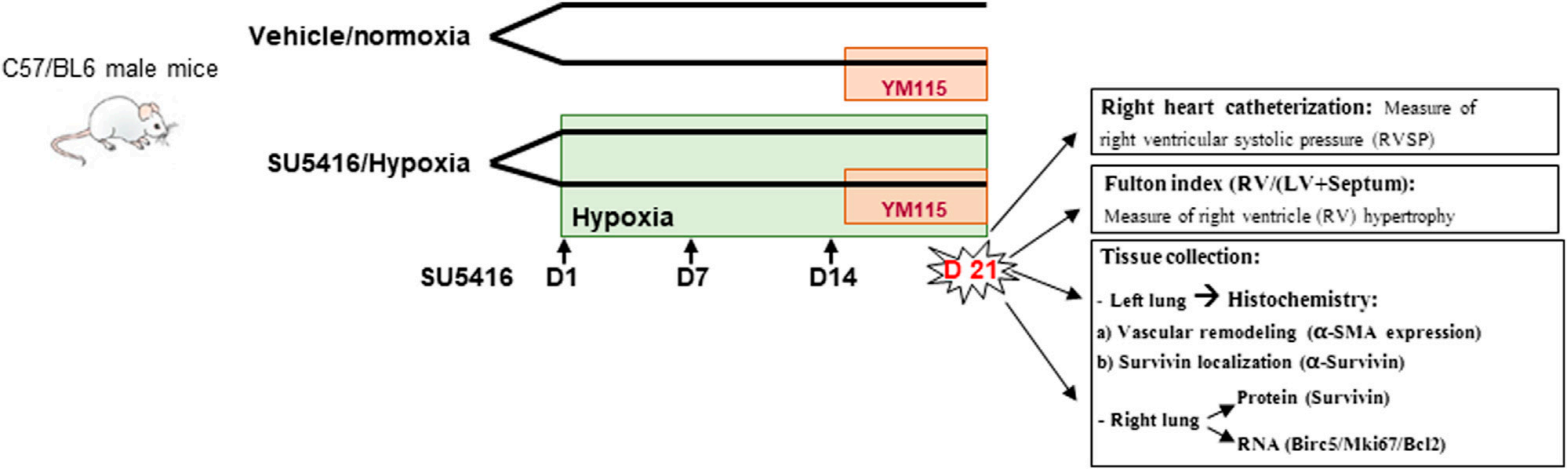
Schematic of the experimental design. Vehicle: DMSO; SU5416: Sugen; YM155: Survivin inhibitor.

In the SU5416/hypoxia model, thirty-two male C57BL/6J mice (Netherlands, Envigo) were divided into four groups: vehicle, normoxia (Veh-Nx); vehicle, normoxia and YM155 (YM155-Nx); SU5416, hypoxia (SU-Hx); and SU5416, hypoxia and YM155 (SU-Hx-YM155). PAH was induced in the SU5416-hypoxia groups by exposure to 10% O_2_ in a normobaric hypoxic chamber (ProOx P360 senses oxygen, Biospherix, NY) plus weekly subcutaneous injections of SU5416 (20 mg/kg in DMSO, Tocris Bioscience, Bristol, United Kingdom) for 3 weeks. Control groups were housed in room air and vehicle (DMSO) was administered each week for 3 weeks. In the groups treated with the survivin inhibitor, 5 mg/kg of YM155 (CAS 781661-94-7, Calbiochem), resuspended in saline solution, was administrated subcutaneously every day during the last week of SU5416/hypoxia exposure (Figure 1). The dose of YM155 used in our study was selected based on the findings of Nakahara et al. (Nakahara et al., 2011).

All animal procedures were approved by the ethics review board on animal research of the University of Barcelona (CEEA 377/13) and complied with national and international guidelines.

We measured right ventricle systolic pressure (RVSP) with a 1F 20 cm catheter inserted through the jugular vein. Afterwards, mice were mechanically ventilated, and a midline sternotomy was performed to expose the heart and lungs. The right lung was excised for gene and protein expression analysis. The left lung was distended until full inflation and fixed by perfusion with 4% phosphate-buffered formaldehyde through the tracheal tube at a constant pressure of 25 cm H_2_O for 24 h. In the excised heart, we calculated the Fulton index (weight of right ventricle (RV)/weight of left ventricle (LV) + septum) to assess right ventricular hypertrophy. Phosphate-buffered formaldehyde-fixed paraffin sections obtained from the midsection of the left lung were used for morphometric analysis. Standard hematoxylin-and-eosin, Von Willebrand Factor and smooth muscle *a*-actin (SMA) stains were performed. More than 100 small vessels (diameter <50 μm) per lung sections were assessed in all the slides. Vessels immunostained for smooth muscle α-actin (SMA) were further classified semi-quantitatively, depending on the proportion of the vessel wall positive for SMA, as: non-muscularized, ≤1/4 of the vessel wall; partially muscularized, >1/4 to 3/4 of the vessel wall; or fully muscularized, >3/4 of the muscle wall. Vascular remodeling was expressed as a percentage of positive immunostaining for SMA (partially muscularized or fully muscularized of the vessel wall) per number of small pulmonary vessels (positive staining for Von Willebrand Factor) in the whole section.

In lung homogenates, we assessed the expression of the BIRC5 gene, which encodes the protein baculoviral inhibitor of apoptosis repeat-containing-5, commonly known as survivin. Survivin inhibits apoptosis and also induces proliferation by increasing mRNA levels of Bcl2, a family of proteins that regulate cell death, by inhibiting apoptosis, and inducing the proliferation marker Mki67. Accordingly, to further characterize the mechanisms of apoptosis and proliferation related to survivin, we examined mRNA expression of *Bcl2*, the founding member of the Bcl2 family, and Mki*67* in the lungs of experimental animals by quantitative RT-PCR.

In the MCT rat model, only survivin and the relationship to MCT-induced changes were assessed. Male Sprague–Dawley rats (*n* = 19) received a single subcutaneous injection of monocrotaline (MCT; 40 mg/kg, Sigma Aldrich) at day 0 to induce PAH. Control group (*n* = 16) received a subcutaneous injection of PBS. RVSP, right ventricular hypertrophy (Fulton Index) and survivin expression protein were measured in groups of control and MCT-treated rats.

### Patients with PAH

Survivin, BCL2 and MKI67 expression were also evaluated in lung samples from patients with PAH and control lungs. Samples of patients with PAH were obtained from explanted lungs provided by the lung transplant unit of Hospital Vall d’Hebron, Barcelona and the Pulmonary Biobank Consortium of the Biomedical Research Networking Center on Respiratory Diseases (CIBERES). Ten lungs from non-smoker, no-cancer donors, discarded to transplant, also provided by the CIBERES Pulmonary Biobank Consortium, were used as controls. The protocol was approved by the institutional review board of Hospital Clínic of Barcelona (CEIm 2013/8571) and that of the Pulmonary Biobank Consortium.

### Statistical analysis

Parametric data are presented as mean ± SD and non-parametric data as median (interquartile range). Groups were compared using, as appropriate, unpaired Student’s t tests, non-parametric Mann-Whitney U test or Kruskal Wallis, or one-way ANOVA with Bonferroni *post hoc* tests using Prism 9 software (GraphPad Prism Software, San Diego, CA). A *p*-value < 0.05 was considered significant in all cases.

## Results

### Survivin expression in experimental PAH

**FIGURE 2:**
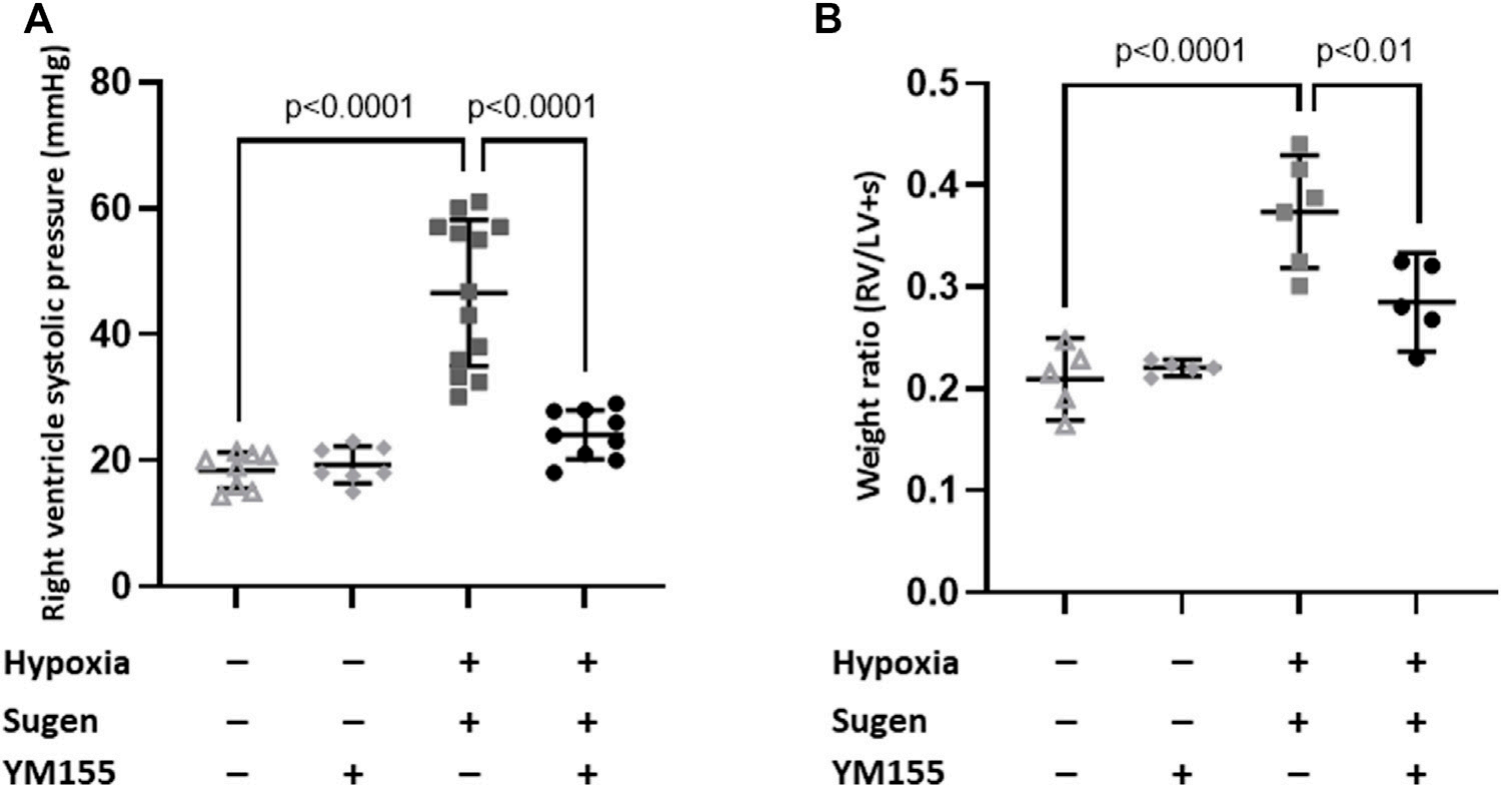
Effect of YM155 on right ventricle systolic pressure and hypertrophy in SU5416/hypoxia-induced PAH mice. Values of right ventricle systolic pressure (RVSP) **(A)** and right ventricular hypertrophy as measured by the Fulton’s index **(B)** in the different study groups. Values are expressed as mean ± SD. Statistical significance was assessed by the one-way ANOVA. Drugs doses used were: SU5416, 20 mg/kg, s. c.i; YM155, 5 mg/kg, s. c.i.

In the SU5416/hypoxia mouse model, SU-Hx mice showed a significant increase in RVSP (46.6 ± 11.6 mmHg), compared with control animals (18.4 ± 2.9 mmHg, *p* < 0.0001) (Figure 2A). These hemodynamic differences were consistent with differences in right ventricular hypertrophy, assessed by the Fulton index (RV/LV + S). RV weight was increased in SU-Hx mice (0.37 ± 0.05) compared with control mice (0.21 ± 0.03; *p* < 0.0001) (Figure 2B).

**FIGURE 3:**
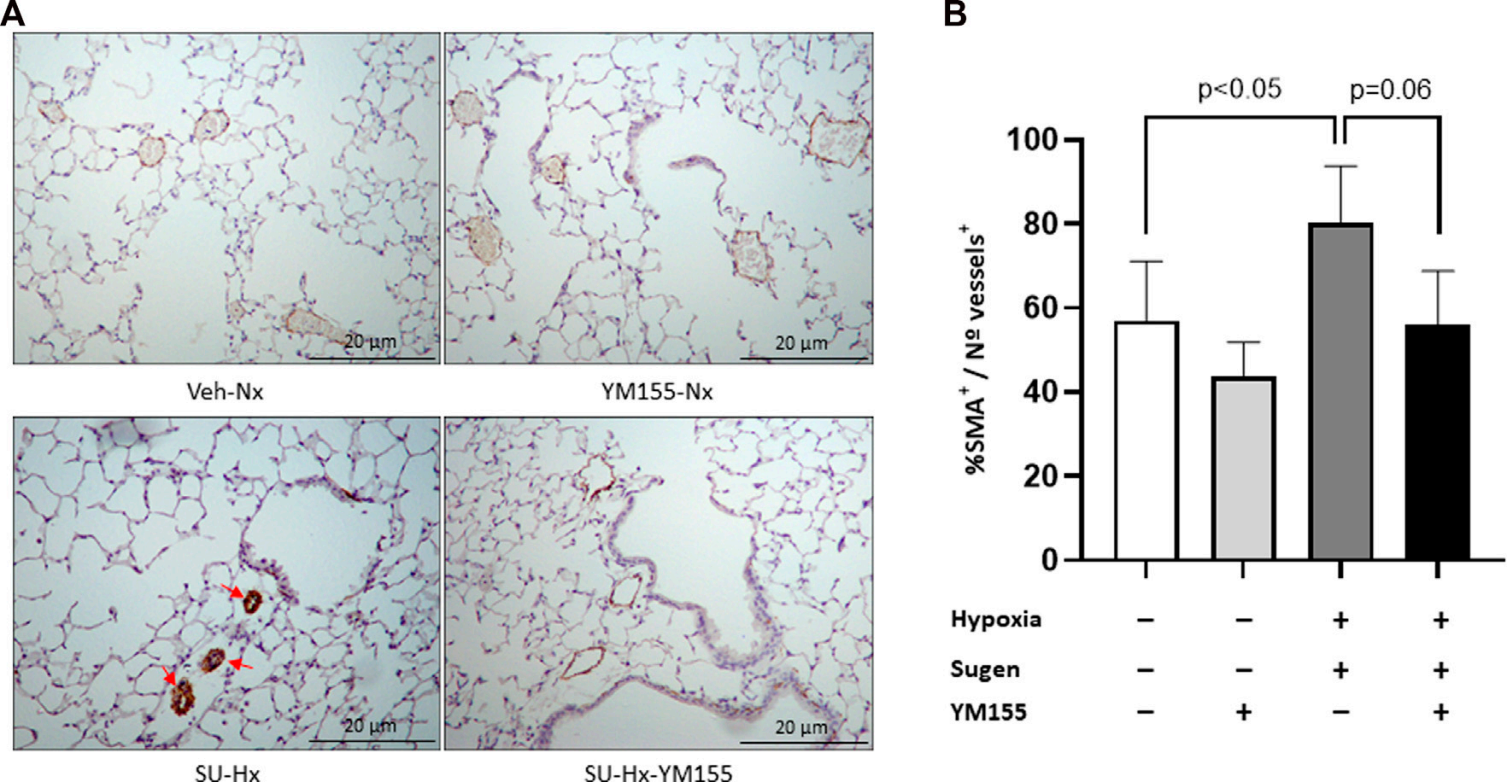
Effect of YM155 on pulmonary vascular remodeling in SU5416/hypoxia-induced PAH mice. **(A)** Representative α-smooth muscle actin (α-SMA) stained pulmonary arterioles in the different experimental groups (Veh-Nx = vehicle and normoxia; YM155-Nx = vehicle, normoxia and YM155; SU-Hx = SU5416 and hypoxia; SU-Hx-YM155 = SU5416, hypoxia and YM155). **(B)** Percent of α-SMA positive arteries with respect to the total number of vessels in each experimental group. Values are expressed as median (IQR) and statistical significance was assessed by the *Kruskal–Wallis* test. Arrows indicate muscularised small pulmonary arteries strongly immunostained with alpha α-SMA antibody.

The SU-Hx group exhibited a higher proportion of muscularized vessels (80.2% ± 6.2% SMA^+^/total number of vessels) compared with controls (Veh-Nx) (57.0% ± 6.6% SMA^+^/total number of vessels; *p* = 0.05) (Figure 3).

**FIGURE 4:**
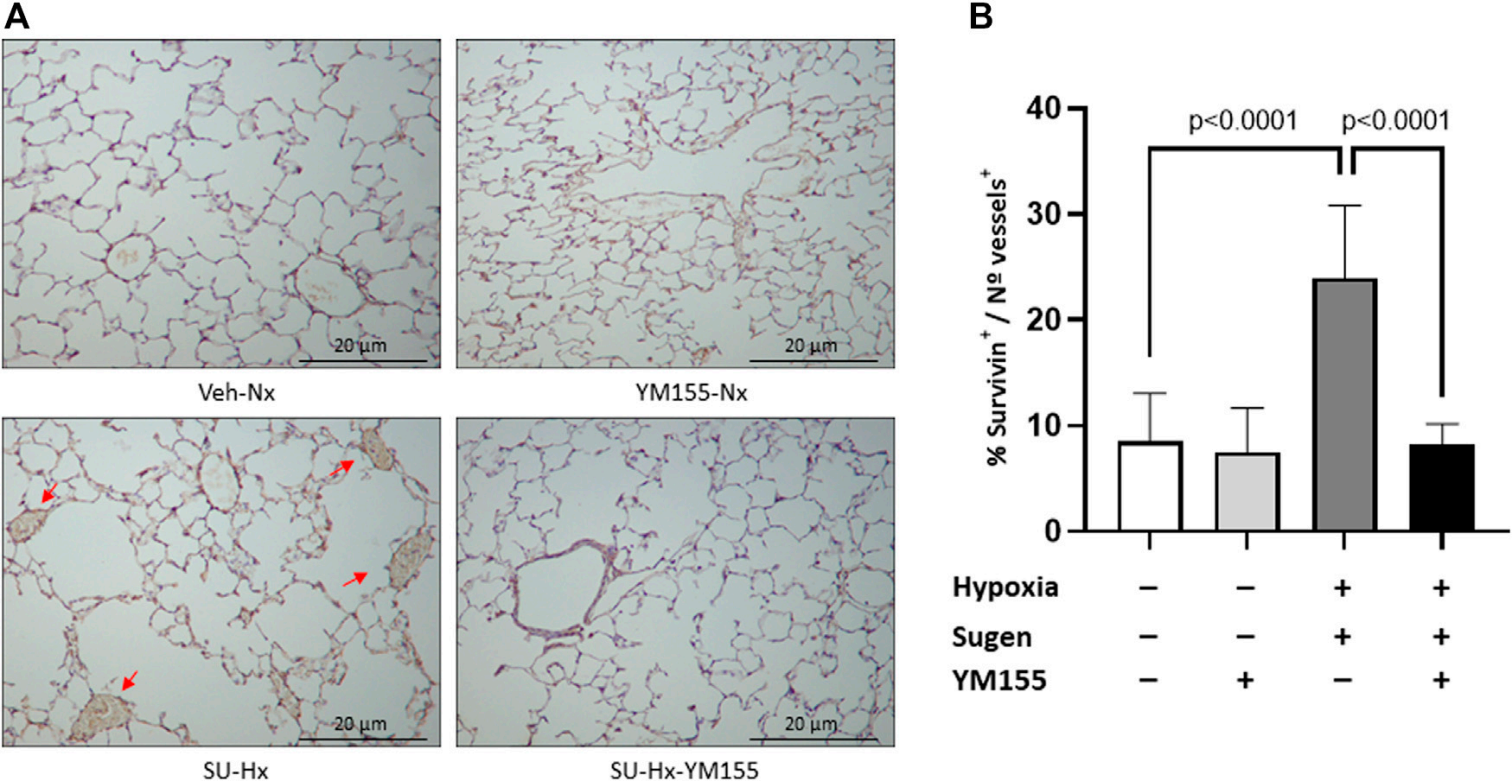
Effect of YM155 on survivin protein expression in small pulmonary arteries in SU5416/hypoxia-induced PAH mice. **(A)** Photomicrographs showing the immunostaining for survivin in small pulmonary vessels in the different experimental groups (Veh-Nx = vehicle and normoxia; YM155-Nx = vehicle, normoxia and YM155; SU-Hx = SU5416 and hypoxia; SU-Hx-YM155 = SU5416, hypoxia and YM155). **(B)** Percent of survivin positive vessels with respect to the total number of vessels in each experimental group. Values are expressed as mean ± SD. Statistical significance was assessed by the one-way ANOVA. Arrows indicate the localization of survivin in small pulmonary arteries.

The immunohistochemical analysis showed an increased expression of survivin in pulmonary vessels of SU-Hx mice (23.9% ± 2.6% survivin^+^ vessels/total number of vessels) compared with the Veh-Nx group (8.5% ± 1.5% survivin^+^ vessels/total number of vessels; *p* = 0.0001) (Figure 4).

**FIGURE 5:**
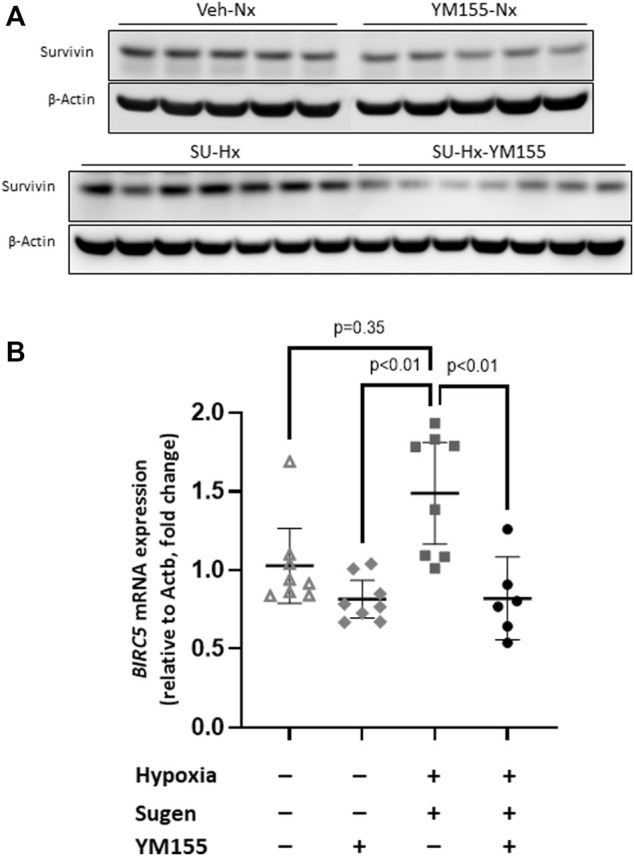
Effect of YM155 on survivin protein and mRNA expression in whole lung extracts of SU5416/hypoxia-induced PAH mice. **(A)** Immunoblots of the expression of survivin and the constititutive protein *ß*-actin in the different experimental groups (Veh-Nx = vehicle and normoxia; YM155-Nx = vehicle, normoxia and YM155; SU-Hx = SU5416 and hypoxia; SU-Hx-YM155 = SU5416, hypoxia and YM155). **(B)** Quantitative mRNA expression of BIRC5, the gene encoding survivin, related to Actb expression using RT-PCR in the different experimental groups. Values are expressed as median (IQR) and statistical significance was assessed by the *Kruskal–Wallis* test.

The expression of survivin protein in whole lung extracts, assessed by western blot analysis, was numerically higher in the SU-Hx group (1.33 ± 0.30) compared with the Veh-Nx group (1.09 ± 0.32) (*p* = 0.07) (Figure 5A).

Quantitative RT-PCR assessment of mRNA survivin levels showed similar results. Survivin gene (BIRC 5) expression was twofold higher in the SU-Hx group than in the Veh-Nx group (*p* = 0.01) (Figure 5B).

**FIGURE 6:**
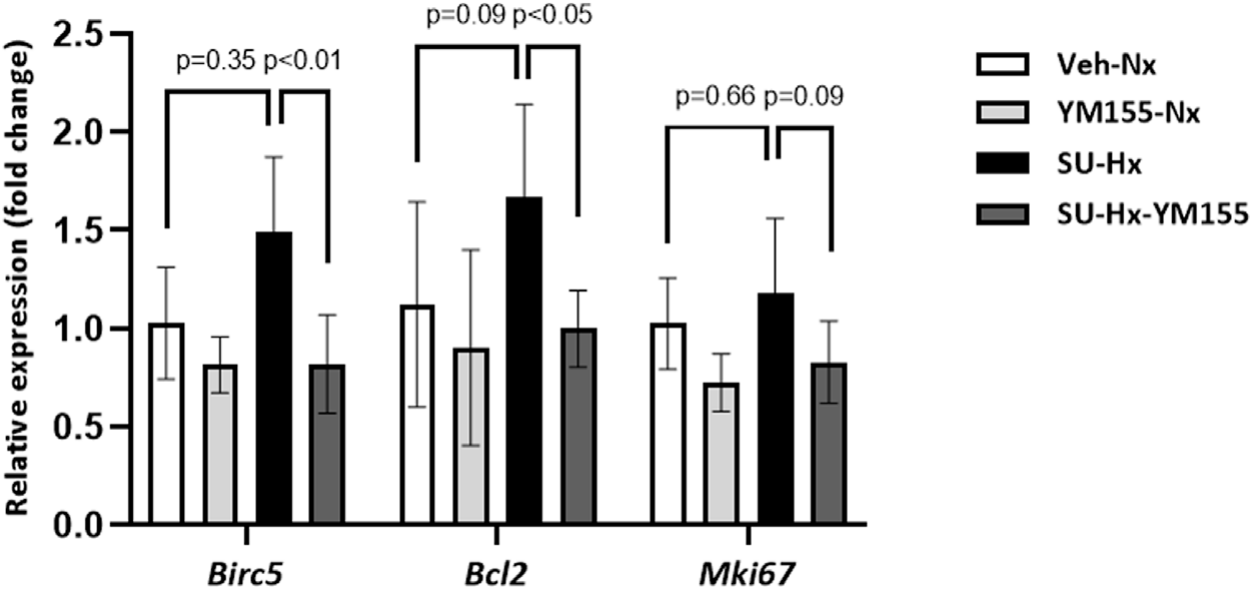
Effect of YM155 on apoptosis/proliferation related genes in whole lung extracts of SU5416/hypoxia-induced PAH mice. Quantitative mRNA expression of *Birc5*, *Bcl2*, and *M*ki*67*, related to Actb expression, using RT-PCR in the different experimental groups. Bcl2 and Mki67 values are expressed as mean ± SD and statistical significance was assessed by the one-way ANOVA. Birc5 values are expressed as median (IQR) and statistical significance was assessed by the *Kruskal–Wallis* test.

mRNA expression in lung homogenates of the apoptosis regulator Bcl2 and the proliferation marker Mki67 were increased in the SU-Hx group, compared with the control Veh-Nx group (*p* < 0.05) (Figure 6).

In the rat model, after MCT administration, RVSP was measured in groups of control and MCT-treated rats (*n* = 2/3/group/week). RVSP was significantly increased in MCT-challenged rats by 2 weeks, rising to a level of 71.5 (±5.6 mmHg), compared to 26.1 (±0.6 mmHg) in controls, by week 4 (Supplementary Figure S1A). Furthermore, a significant elevation in right ventricular hypertrophy (Fulton Index) was observed after 3-week MCT treatment (Supplementary Figure S1B). We assessed the expression of survivin protein in the rat lung, survivin gene expression was also twofold higher in MCT rats (*p* < 0.001) compared to controls (Supplementary Figure S2).

### Effects of treatment with YM155 in the SU5416/hypoxia mouse model

In the mice model, treatment with YM155 significantly reduced RVSP (24.1 ± 3.9 mmHg) (*p* < 0.0001 compared with SU-Hx) (Figure 2A) and attenuated RV hypertrophy in SU-Hx mice (0.28 ± 0.04, *p* = 0.01) compared with untreated SU-Hx mice) (Figure 2B).

The proportion of vessels with muscularized walls trended to be lower in the SU-Hx-YM155 group (55.9% ± 3.2% SMA^+^/total number of vessels) compared with the SU-Hx group (*p* = 0.06), with values similar to those of the Veh-Nx group (Figure 3).

Additionally, treatment with YM155 significantly decreased survivin expression in SU-Hx mice (8.3% ± 0.7% survivin^+^ vessels/total number of vessels; *p* = 0.0001 compared with untreated SU-Hx mice) (Figure 4) and also reduced survivin protein expression in SU-Hx mice (0.57 ± 0.21) (*p* = 0.0012, compared with the SU-Hx untreated group) (Figure 5A). Additionally, treatment with YM155, reduced survivin mRNA expression in SU-Hx mice (*p* = 0.01, compared with the untreated SU-Hx group) to levels equivalent to those in the Veh-Nx group (Figure 5B).

In terms of apoptosis/proliferation markers, treatment with YM155 significantly also decreased the expression of both Bcl2 and Mki67 genes in SU-Hx mice (*p* < 0.05, compared with the SU-Hx untreated group) (Figure 6).

### Survivin and apoptosis/proliferation axis-related genes expression in patients with PAH

**FIGURE 7:**
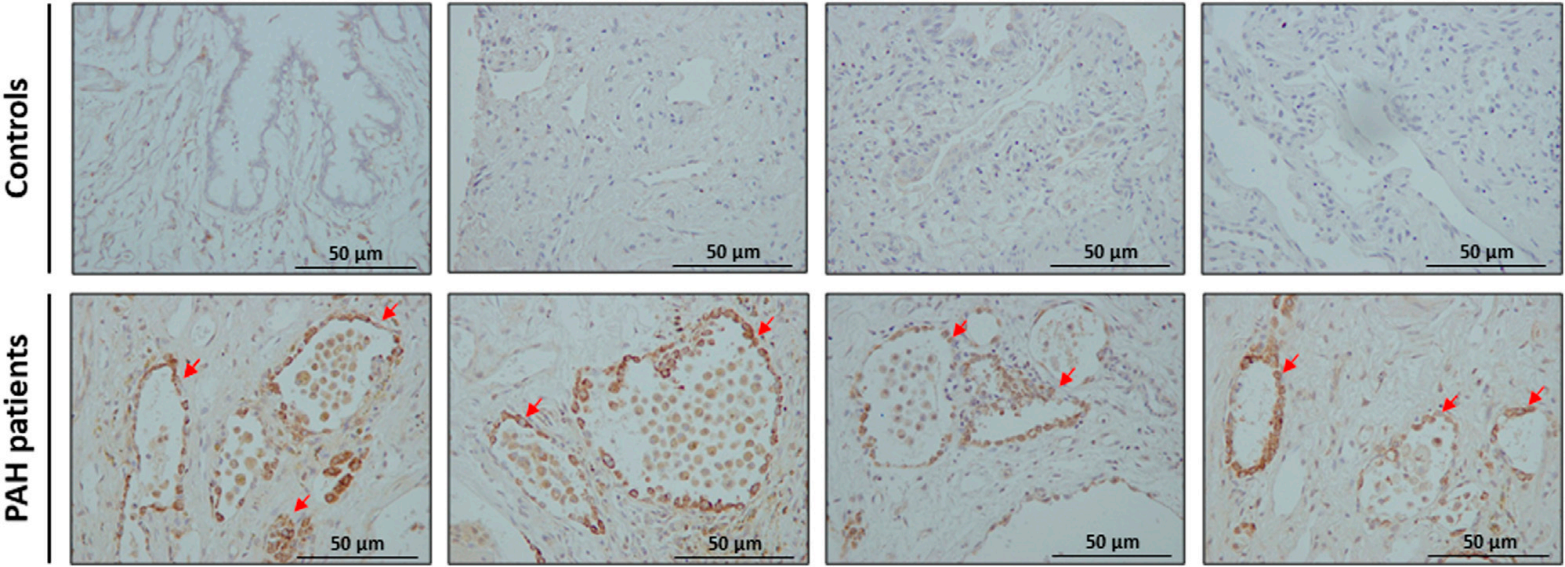
Expression of survivin in pulmonary arteries of patients with PAH. Representative photomicrographs showing immunohistochemical staining and labelling with anti-survivin antibody in pulmonary arteries of patients with PAH but not in lungs of control individuals. Arrows indicate survivin positive cells in pulmonary arteries.

Immunohistochemical analysis of explanted lungs showed prominent expression of survivin in the intimal layer of pulmonary arteries of patients with PAH, whereas it was not expressed in pulmonary arteries of control lungs (Figure 7).

**FIGURE 8:**
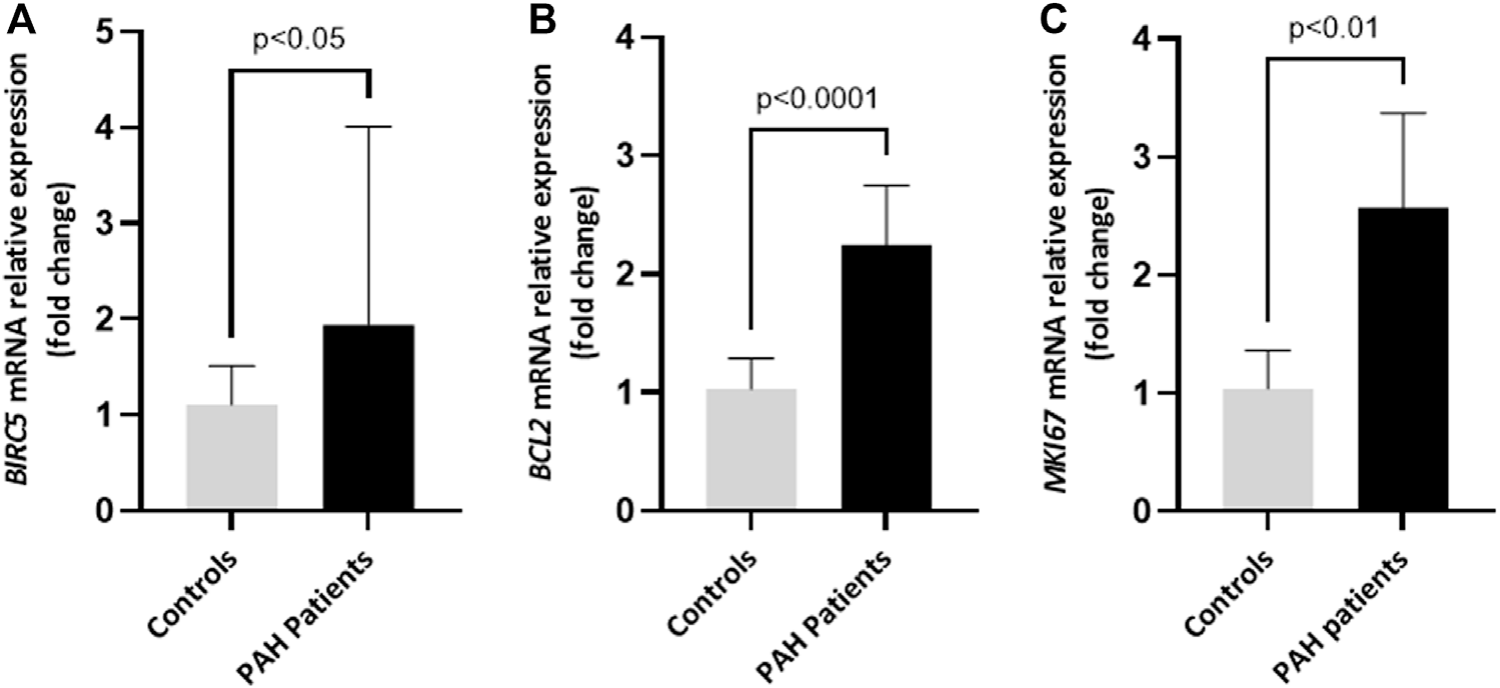
mRNA expression of survivin and genes involved in apoptosis and proliferation in whole lung extracts of patients with PAH. Quantitative RT-PCR assay of the mRNA expression of *BIRC5*, the gene encoding survivin **(A)**, *BCL2* **(B)** and *MKI67* **(C)** in lung explants from patients with PAH compared with control lungs. *BIRC5* values are expressed as median (IQR) and statistical significance was assessed by the U-Mann Whitney test. *BCL2* and *MKI67* values are expressed as mean ± SD and statistical significance was assessed by the two-tailed Student´s t-test.

Quantitative RT-PCR analysis confirmed increased mRNA expression of BIRC5, the gene encoding survivin, in lungs of patients with PAH, compared with control subjects (*p* < 0.05) (Figure 8A). Patients with PAH also showed increased lung expression of BCL2 and MKI67 genes compared with control lungs (*p* < 0.0001) (Figures 8B, C).

## Discussion

Results of the present study show that the expression of the anti-apoptotic protein survivin is increased in pulmonary arteries of patients with PAH and in the lungs of two experimental models of PAH, the SU5416/hypoxia mouse and the MCT rat. In the SU5416/hypoxia model, treatment with the survivin inhibitor YM155 prevented the development of PH, reduced the transcriptional and post-transcriptional expression of survivin and also that of genes related with apoptosis/proliferation pathways, which can be understand as a positive attribute with a consistent translational relevance.

In our study we show increased transcriptional and post-transcriptional expression of survivin in pulmonary arteries and lungs of patients with PAH and also in two validated experimental models of PAH (Blanco et al., 2017; Bueno-Beti et al., 2018). The increased expression of survivin was associated with a disbalanced expression of genes related with apoptosis and cell proliferation, suggesting a mechanistic role of survivin, an anti-apoptotic molecule, in the development of PAH and their consequences on the right ventricle. Our findings concur with those of McMurtry et al. (McMurtry et al., 2005), who showed increased survivin expression in pulmonary arteries of 6 patients with PAH and in the MCT-induced PH rat model, and with those of other models *in vitro* and *in vivo* exposed to hypoxia (Fan et al., 2015; Zhang et al., 2015; Zhang et al., 2016; Ye et al., 2022). We also extend these results to the SU5416-hypoxia mouse model and show the interaction between survivin expression and genes related with apoptosis/proliferation pathways. Overall, these findings suggest that survivin may contribute to the antiapoptotic, proproliferative cell phenotype that characterizes PAH (Humbert et al., 2019).

Treatment of SU5416/hypoxia mice with the survivin inhibitor YM155 (sepantronium bromide) reduced the expression of survivin, both at the transcriptional and post-transcriptional levels, in the whole lung and in the pulmonary artery wall. Importantly, SU5416/hypoxia mice treated with YM155 did not develop pulmonary vascular remodeling, increase of RVSP or right ventricular hypertrophy. Similar findings were observed in high blood flow-induced PAH and chronic hypoxia models (Fan et al., 2015; Zhang et al., 2015; Zhang et al., 2016; Ye et al., 2022) and also using gene therapy, inhalation of an adenovirus carrying a phosphorylation-deficient survivin mutant with dominant-negative properties, in the monocrotaline-induced PH rat model by McMurtry et al. (McMurtry et al., 2005). That said, our study adds information to a model that is closer to the HAP. That said, our study shows that treatment with YM155 leads to reduced expression of survivin in a model that closely mimics HAP. All in all, these observations suggest that survivin inhibition might conform a novel potential therapeutic approach to PAH, which is currently feasible with the use of YM155.

The balance between apoptosis and cell proliferation related genes was altered in the lungs of PAH patients and also in the SU5416/hypoxia mouse model, with an increased expression of pro-proliferative genes, such BCL2 and MKI67. The vascular endothelial growth factor (VEGF) is an important angiogenic mediator and a key factor for endothelial cell survival, due to its anti-apoptotic effect (Voelkel and Gomez-Arroyo, 2014). VEGF expression levels are increased in some types of tumors and in patients with PAH (Tuder et al., 2013). We also observed an increased expression of VEGF in endothelial and smooth muscle cells and in fibroblasts of mice with SU5416/hypoxia-induced PH (data not shown). VEGF enhances endothelial cell survival by inducing the expression of Bcl2 through the activation of extracellular signal-regulated kinases (ERK) 1/2 and phosphoinositide 3-kinase (PI3-K)/protein kinase B (Akt) signaling pathways (Zhao et al., 2011). Both ERK and Akt play an important role in the proliferation, differentiation and survival of different cell types and have been related to a wide variety of anti-apoptotic functions (Kumar et al., 2004; Kovacs et al., 2019). Furthermore, BCL2 inhibits cell death signals in endothelial cells through the upregulation of survivin (Kumar et al., 2004; Li et al., 2008). Interestingly, treatment with YM155 not only reduced the expression of survivin but also that of Bcl2 and the proliferation of SMA + cells in pulmonary arteries of SU5416/hypoxia-exposed mice, suggesting that it restored the proliferation/apoptosis balance and prevented pulmonary vascular remodeling.

The therapeutic efficacy and safety of the survivin inhibitor YM155 has been evaluated in phase I and II clinical trials in different types of cancers (Giaccone et al., 2009; Miura et al., 2011) and in on-going trials registered in clinicaltrials.gov. Trials reported so far have shown adequate tolerability of YM155 and antitumoral activity in different types of tumors (Tolcher et al., 2012; Kelly et al., 2013). Therefore, if there are no adverse safety signals in the ongoing clinical studies, the effect of YM155 could be evaluated in patients with PAH to assess its potential usefulness.

Our study has strengths and limitations. The robust effect of the inhibitor on hemodynamic and histologic parameters of experimental PAH *in vivo* and the inclusion of lung explant samples are strengths of the study. There are also some limitations; first, the assessment of the effects of YM155 in the experimental model used a “preventive” design. That is, YM155 was administered during the last week of the development of the experimental model. Therefore, we cannot assert that YM155 will have the same effect when lung lesions are already developed, which is what happens in patients with PAH.

Second, YM155 was used only in an animal model, but the results are similar to those of McMurtry *et al*. (McMurtry et al., 2005) using gene therapy and other recent studies (Fan et al., 2015; Zhang et al., 2015; Zhang et al., 2016; Ye et al., 2022). Therefore, the concept of the beneficial effect of inhibition of survivin in experimental PAH is well demonstrated, and now in this study in a more robust experimental model than that of rat exposed to monocrotaline or hypoxia. Third, mice were treated with the half maximum tolerated dose of YM155 but dose optimization was not performed in the animal model and no drug concentrations were assessed.

Finally, the increased expression of survivin in pulmonary arteries was shown in patients with end-stage PAH, undergoing lung transplantation. Therefore, we cannot conclude that the same will occur in patients with less severe PAH.

In conclusion, survivin expression is increased in pulmonary arteries of patients with PAH and in a robust experimental model of PAH, suggesting that it might be involved in the pathogenesis of PAH by disturbing apoptosis/proliferation signaling pathways. In experimental PAH, treatment with the survivin inhibitor YM155 prevented the development of pulmonary vascular remodeling and hence that of PH and its consequences on the right ventricle by restoring the balance between genes related to apoptosis and cell proliferation. Accordingly, survivin inhibition with YM155 might represent a novel therapeutic target for this devasting disease that deserves further evaluation.

## Data Availability

The original contributions presented in the study are included in the article/Supplementary Material, further inquiries can be directed to the corresponding author.

## Abbreviations

Akt, protein kinase B; EC, endothelial cells; ERK, extracellular signal-regulated kinases; LV, left ventricle; MCT-PAH, monocrotaline-induced PAH model; PAH, pulmonary arterial hypertension; PASMC, pulmonary artery smooth muscle cells; PI3-K, phosphoinositide 3-kinase; RV, right ventricle; RVSP, right ventricle systolic pressure; SMA, smooth muscle α-actin; SU-Hx, SU5416, hypoxia; SU-Hx-YM155, SU5416, hypoxia and YM155; VEGF, vascular endothelial growth factor; Veh-Nx, vehicle, normoxia; YM155, sepantronium bromide; YM155-Nx, vehicle, normoxia and YM155.
