# Supplementary material for: Survivin inhibition with YM155 ameliorates experimental pulmonary arterial hypertension
Source: Front Pharmacol. 2023 Apr 24;14:1145994. doi: 10.3389/fphar.2023.1145994 (PMC10176173; doi:10.3389/fphar.2023.1145994)
Supplement: Supplementary file 1 [file DataSheet1.doc]

**Supplemental material**

**MATERIALS AND METHODS**

***In vivo pulmonary hypertension mouse model***

Thirty-two male C57BL/6J mice (Netherlands, Envigo) were divided into four groups: vehicle, normoxia (Veh-Nmx); vehicle, normoxia and YM155 (YM155-Nmx); Su5416, hypoxia (SU-Hx); and Su5416, hypoxia and YM155 (SU-Hx-YM155). PAH was induced in the Su5416 hypoxia groups by exposure to 10% O_2_ in a normobaric hypoxic chamber equipped with oxygen (ProOx P360 senses oxygen, Biospherix, NY) and temperature sensors plus weekly subcutaneous injections of Su5416 (20 mg/kg in DMSO, Tocris Bioscience, Bristol, UK) for three weeks. Control groups were hosed in room air and vehicle (DMSO) was administered each week for three weeks. In the groups treated with survivin inhibitor, 5 mg/kg of YM155 (CAS 781661-94-7 – Calbiochem), resuspended in saline solution, was administrated subcutaneously every day during the last week of Su5416/hypoxia exposure (Figure 1). The dose of YM155 used in our study was selected based on the findings of Nakahara et al. (1).

All animal procedures were approved by the ethics review board on animal research of the University of Barcelona (CEEA 377/13) and complied with national and international guidelines.

***Measurements of right ventricular pressure and ventricular weight***

Three weeks after last treatment, animals were anesthetized by intraperitoneal injection of ketamine:xylazine (80-100 mg/kg:10-16 mg/kg). A SPR-1000 Mouse Pressure Catheter (Millar, Inc., Houston, Texas 77023 USA) was inserted from the right jugular vein, and right ventricular pressure was measured and analysed by LabChart v6.1.3 software (ADInstruments Ltd., OX4 6HD United Kingdom). In order to evaluate right ventricular hypertrophy, right ventricle (RV) was dissected from the left ventricle (LV) including septum (S), and the RV/LV+S weight ratio and RV/body weight ratio were calculated (2,3).

***Tissue preparation***

After hemodynamic measurements, the mice were mechanically ventilated, and a midline sternotomy was performed to expose the heart and lungs. The right peripheral lung sections were excised for gene and protein expression analysis. The left lungs were distended until fully inflated and fixed by perfusion at 25 cm H2O column constant pressure for 24h through a tracheal tube with 4% phosphate-buffered formaldehyde. Phosphate-buffered formaldehyde-fixed paraffin sections obtained from the midsection of the left lung were used for morphometric analysis. More than 100 small vessels (diameter <50 μm) per lung sections were assessed in all the slides.

***Morphometric analysis***

Left lung tissues were dissected in three 2,0 mm transversal sections and paraffin embedded, 2-4 μm lung sections were standard haematoxylin-and-eosin staining and Von Willebrand Factor staining were performed and analysed for evidence number of small intrapulmonary vessels (diameter <50 μm). Intrapulmonary vessels immunostained for smooth muscle α-actin (SMA) were further classified semi-quantitatively, depending on the proportion of the vessel wall positive for SMA: non-muscularised ≤1/4 of the vessel wall; partially muscularised >1/4 to ≤3/4 of the vessel wall; or fully muscularised >3/4 of the muscle wall. Vascular remodelling was expressed as a percentage of positive immunostaining for SMA (partially muscularised or fully muscularised of the vessel wall) per number of small intrapulmonary vessels (positive staining for Von Willebrand Factor) in the whole section.

***Immunohistochemistry***

Immunohistochemistry was performed using the Envision Dual Link System-HRP (DAB+) Kit from Dako (Cat. No. K4007, Dako, Carpinteria, CA, USA). Paraffin-embedded lung tissue slides were deparaffinized and hydrated. Epitope retrieval was performed by boiling the sections in citrate buffer (0.01 M, pH 6.0). Endogenous peroxidase activity was quenched with Peroxidase Blocking Reagent. After blocking in 1% BSA plus 4% normal goat serum (Cat. No. G9023, Sigma/Aldrich), the sections were stained with mouse anti-human smooth muscle actin (1:100 dilution, clone 1A4, Cat. No. M0851, Dako), rabbit anti-Survivin (diluted 1:2000, Cat. No. Ab469, Abcam) and rabbit anti-Von Willebrand Factor (diluted 1:1000, Cat. No. Ab6994, Abcam) antibodies. These sections were incubated with primary antibodies overnight at 4°C. After applying a secondary- HRP-conjugate, the slides were incubated with 3,3’diaminobenzidine substrate (DAB) and counterstained with haematoxylin. The pictures were taken with a Leica CTR5000 microscope system (Leica Microsystems GMBH, Wetzlar Germany).

***Quantitative RT-PCR***

Human *BIRC5* (survivin) gene expression assay was performed according to the following protocol: Total RNA from human lungs was extracted with the miRNeasy Mini Kit (Qiagen, Hilden, Germany), according to the supplier’s instructions. A NanoDropTM 1000 Spectrophotometers (Thermo Scientific, US) was used to determine the final RNA amount and quality. cDNA was synthesized from one μg of total RNA primed with a random hexamer using iScriptTM cDNA Synthesis Kit (Biorad, USA). Real-Time PCR Assays using TaqMan probes (Applied Biosystems, USA) for the specific detection of *BIRC5* was performed utilizing a 7.900HT Fast Real-time PCR System (Genomic Centre, Complutense University of Madrid, Spain). Expression of *BIRC5* was normalised to Eukaryotic 18S rRNA (18S) Hs03003631_g1 expression, as endogenous housekeeping gene. Relative gene expression was analysed using the 2−ΔΔCT method (4).

Testing of human *BCL2* and *MKI67* and mice *BIRC5, BCL2, MKI67* gene expression was carried out as follow: Total RNA was extracted from lung tissue using trizol (Bioline, UK) according to the manufacturer’s instructions. cDNA was synthesized from 1,0 μg of total RNA primed with a random hexamer using high-capacity cDNA kit (Applied Biosystems, USA). SYBR Green based quantitative PCR was performed using ViiA 7 Real-Time PCR System (Applied Biosystems Corp., USA). All primers were obtained from Integrated DNA Technologies, BVBA (Leuven, Belgium). For human, the follow sequences of PCR primers were used: BCL2 (Forward: *5’-GGGAGGATTGTGGCCTTCTT-3’*; Reverse: *5’-CAGGTACTCAGTCATCCACA-3’*), MKI67 (Forward: *5’-GCAGCCTTAACTGTGACACTTGC-3’*; Reverse: *5’-GCCACCGTGCCCTGG-3’*). For mice, the follow sequences of PCR primers were used: *BIRC5* (Forward: *5’-AGAGAGCCAAGAACAAAATTGC-3’*; Reverse: *5’-TCTCAGCAAAGGCTCAGCA-3’*), BCL2 (Forward: *5’-TCTTTTCGGGGAAGGATGGC-3’*; Reverse: *5’-TCTCCAGCATCCCACTCGTA-3’*), MKI67 (Forward: *5’-CCTGCCCGACCCTACAAAAT-3’*; Reverse: *5’-TGCTGCTTCTCCTTCACTGG-3’*). Expression of genes was normalised to human GAPDH (Forward: *5’-CATCACCATCTTCCAGGAGC-3’*; Reverse: *5’-TGGACTCCACGACGTACTCA-3’*) and mouse Actb (Forward: *5’-GGAGGGGGTTGAGGTGTT-3’*; Reverse: *5’-GTGTGCACTTTTATTGGTCTCAA-3’*) expression, as endogenous housekeeping genes. Relative gene expression was analysed using the 2−ΔΔCT method (4).

***Western blot analysis***

Lung tissue was homogenized in lysis buffer [RIPA buffer (Cat. No. 89901, Thermo Scientific™) plus Halt™ Protease and Phosphatase Inhibitor Cocktail (Cat. No. 78441, Thermo Scientific™)]. Fifty micrograms of total protein, determined by using a Pierce BCA protein assay kit (Cat. No. 23227, Thermo Scientific), was electrophoresed under reducing conditions by SDS-PAGE on NuPAGE™ 4-12% Bis-Tris Protein Gels (Cat. No. NP0322BOX, Invitrogen) and transferred to a PVDF membrane by use of iBlot2 Transfer Stacks (Invitrogen, Carlsbad, CA). After blocking with TBST plus 5% non-flat milk, membranes were incubated with primary antibodies, including anti-survivin (diluted 1:1000, Cat. No. Ab469, Abcam), anti-β-actin (diluted 1:2000, Cat. No. NB600-503SS, Novus) and anti-GAPDH (diluted 1:2500, Cat. No. ab9485, Abcam) at 4°C overnight. The membrane was incubated with horseradish peroxidase-conjugated secondary antibodies at room temperature for 1 hour. A WesternBright™ Quantum detection kit (Cat. No. K-12042-D20, Advansta) was used to detect bands, which were quantified by use of an Image Quant LAS4000 system (GE Healthcare Bio-Sciences, Sweden) and ImageLab 6.0 software (Bio-Rad Laboratories, Inc., California USA).

***In vivo pulmonary hypertension rat model***

Male Sprague–Dawley rats (n=19) weighting approximately 200 g received a single subcutaneous injection of monocrotaline (MCT; 40 mg/kg, Sigma Aldrich) at day 0 to induce PAH. Control group (n=16) received a subcutaneous injection of PBS. Right ventricular pressures and volumes were recorded using a Millar SPR-869 pressure-volume catheter (Millar Instruments, Houston, TX, USA) in 3 rats of each group at week 1, 2, 3 and 4 in order to identify the onset of the disease. Organs were removed and snap-frozen in liquid nitrogen for RNA and protein isolation and stored at −80°C until further use. Blood samples were taken weekly for a period of 4 weeks.

We also assessed the expression of survivin protein in the rat lung according to the same protocol used for the mouse model. Expression of *survivin* was normalised to GAPDH expression, as endogenous housekeeping gene.

This animal model in rat was developed in the UK, through a collaboration with Cambridge University. Studies were conducted in accordance with the UK Animals (Scientific Procedures) Act 1986 and approved under Home Office Project License 80/2460.

***Subjects***

Survivin expression was also evaluated in biological samples from PAH patients who had undergone lung transplantation and compared with control lungs (donor lungs). The protocol was approved by an institutional review board (CEIm HCB 2013/8571) and was authorised by the biobank ethics committee.

*For immunohistochemistry assay and for gene expression assay:* Eleven males and females (n=11) PAH patients aged between 25 and 57 years at diagnosis with idiopathic PAH (n=4), CTD-PAH (n=4), CHD-PAH (n=2), PVOD (n=1) were studied. Lung samples were obtained from explanted lungs during lung transplantation (Hospital Vall d’Hebron, Barcelona and Hospital 12 de Octubre, Madrid, both in Spain) and stored in the Pulmonary Biobank Consortium.

Ten no-smokers and no-cancer donors, discarded to transplant were used as controls (Centro de Investigación Biomédica en Red de Enfermedades Respiratorias (CIBERES) Biobank).

***Statistical analysis***

Parametric data are presented as mean±SD and nonparametric data as median (interquartile range). Groups were compared using, as appropriate, unpaired Student's t tests, non-parametric Mann-Whitney U test or Kruskal Wallis, or one-way ANOVA with Bonferroni post hoc tests using Prism 9 software (GraphPad Prism Software, San Diego, CA).  A p-value <0.05 was considered significant in all cases.

**References**

1. Nakahara T, Kita A, Yamanaka K, Mori M, Amino N, Takeuchi M, et al. Broad spectrum and potent antitumor activities of YM155, a novel small-molecule survivin suppressant, in a wide variety of human cancer cell lines and xenograft models. Cancer Sci. 2011;102(3):614–21.

2. Nandi M, Miller A, Stidwill R, Jacques TS, Lam AAJ, Haworth S, et al. Pulmonary hypertension in a GTP-cyclohydrolase 1-deficient mouse. Circulation. 2005;111(16):2086–90.

3. Nakanishi N, Ogata T, Naito D, Miyagawa K, Taniguchi T, Hamaoka T, et al. MURC deficiency in smooth muscle attenuates pulmonary hypertension. Nat Commun. 2016;7:12417.

4. Livak KJ, Schmittgen TD. Analysis of relative gene expression data using real-time quantitative PCR and the 2-ΔΔCT method. Methods. 2001;25(4):402–8.

**Figure legends**

**Figure Supplemental 1. Monocrotaline-PAH rat model.**

Rats were administered vehicle or monocrotaline (MCT; 40 mg/kg). (Panel A) Right ventricular systolic pressure (RVSP) and (Panel B) right ventricular hypertrophy (RV/(LV1S)) were assessed in control (circles) and MCT-treated rats (squares) at weeks 1, 2, 3, and 4. Statistical significance was assessed by one-way ANOVA; *p<0.05, ***p<0.001, ****p<0.0001.

**Figure Supplemental 2.** **Monocrotaline-PAH rat model.**

Quantitative mRNA expression of survivin related to GAPDH expression using RT-PCR in the different experimental groups. Statistical significance was assessed by one-way ANOVA; **p<0.01, ***p<0.001
