# Supplementary figures and images for: Survivin inhibition with YM155 ameliorates experimental pulmonary arterial hypertension
Source: Front Pharmacol. 2023 Apr 24;14:1145994. doi: 10.3389/fphar.2023.1145994 (PMC10176173; doi:10.3389/fphar.2023.1145994)

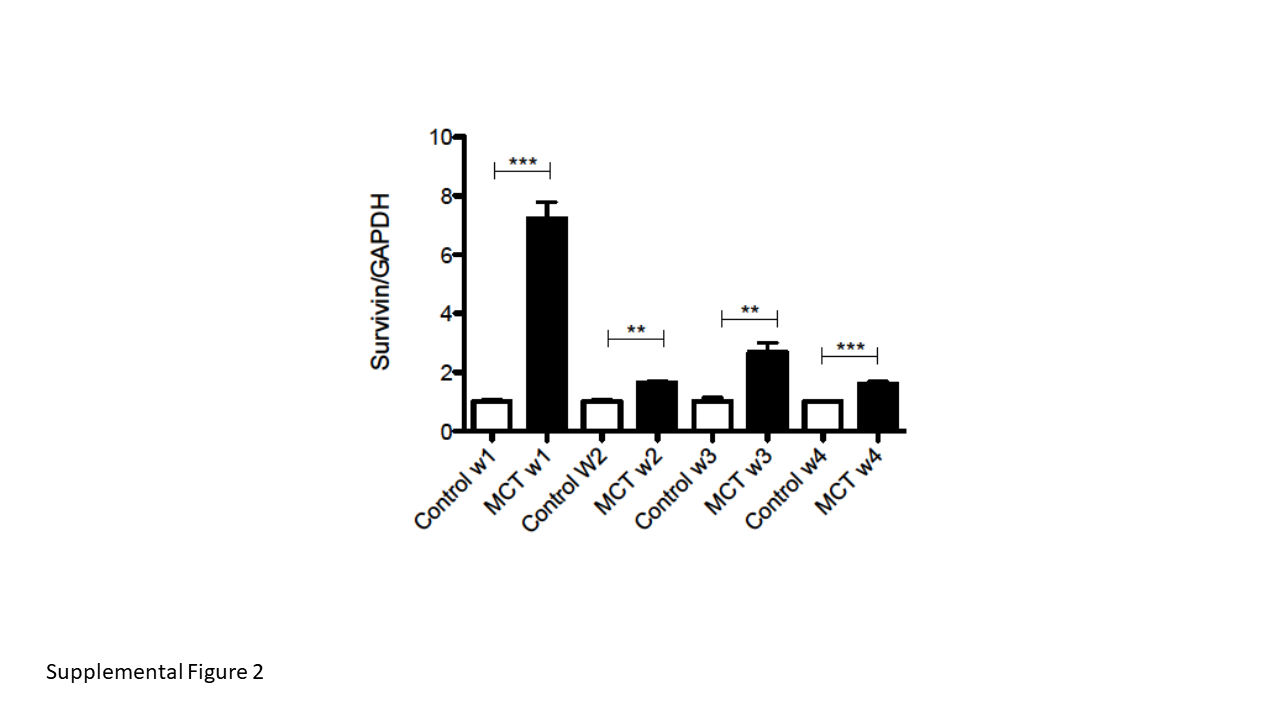

Supplement: Supplementary file 2 [file Image2.TIF]

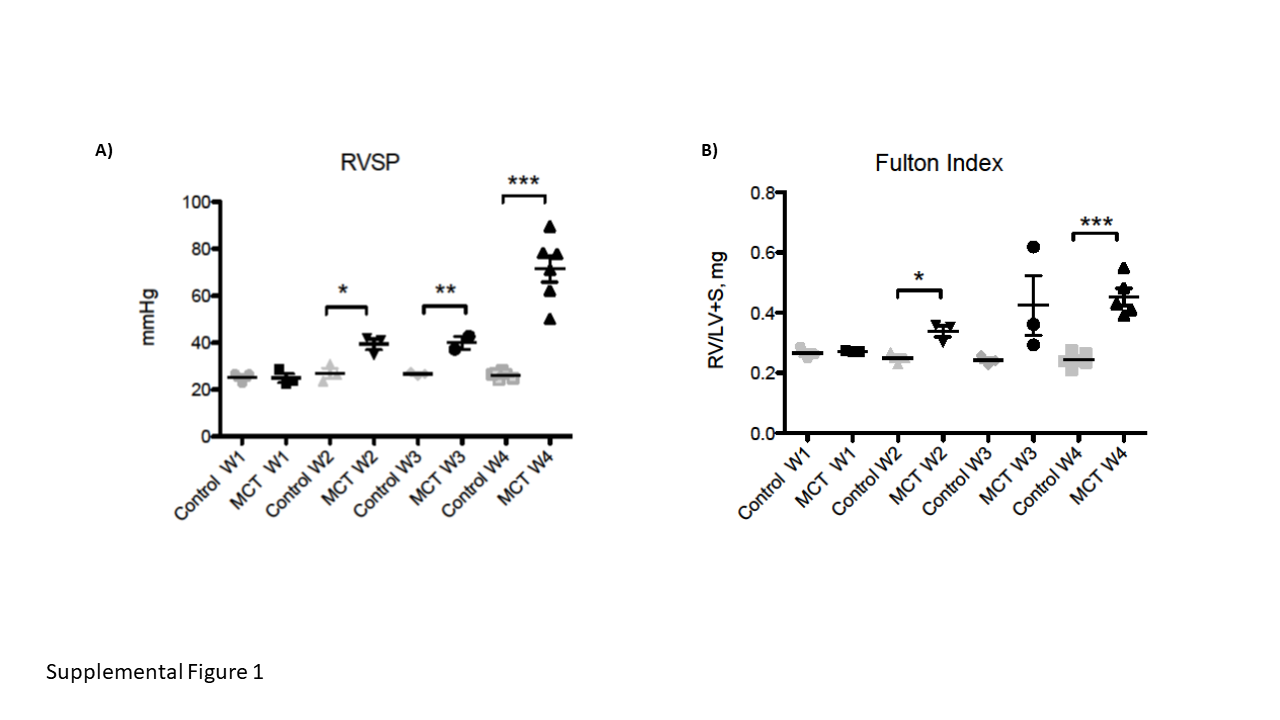

Supplement: Supplementary file 3 [file Image1.TIF]
